# Naringenin potentiates anti-tumor immunity against oral cancer by inducing lymph node CD169-positive macrophage activation and cytotoxic T cell infiltration

**DOI:** 10.1007/s00262-022-03149-w

**Published:** 2022-01-19

**Authors:** Sho Kawaguchi, Kenta Kawahara, Yukio Fujiwara, Koji Ohnishi, Cheng Pan, Hiromu Yano, Akiyuki Hirosue, Masashi Nagata, Masatoshi Hirayama, Junki Sakata, Hikaru Nakashima, Hidetaka Arita, Keisuke Yamana, Shunsuke Gohara, Yuka Nagao, Manabu Maeshiro, Asuka Iwamoto, Mayumi Hirayama, Ryoji Yoshida, Yoshihiro Komohara, Hideki Nakayama

**Affiliations:** 1 Department of Oral and Maxillofacial Surgery, Faculty of Life Sciences, Kumamoto University, Honjo 1-1-1, Chuo-ku, Kumamoto, 860-8556 Japan; 2 Department of Cell Pathology, Graduate School of Medical Sciences, Kumamoto University, Honjo 1-1-1, Chuo-ku, Kumamoto, 860-8556 Japan

**Keywords:** CD169, Regional lymph node, Sinus macrophage, Oral squamous cell carcinoma (OSCC), Naringenin, Anti-tumor immunity

## Abstract

The CD169^+^ macrophages in lymph nodes are implicated in cytotoxic T lymphocyte (CTL) activation and are associated with improved prognosis in several malignancies. Here, we investigated the significance of CD169^+^ macrophages in oral squamous cell carcinoma (OSCC). Further, we tested the anti-tumor effects of naringenin, which has been previously shown to activate CD169^+^ macrophages, in a murine OSCC model. Immunohistochemical analysis for CD169 and CD8 was performed on lymph node and primary tumor specimens from 89 patients with OSCC. We also evaluated the effects of naringenin on two murine OSCC models. Increased CD169^+^ macrophage counts in the regional lymph nodes correlated with favorable prognosis and CD8^+^ cell counts within tumor sites. Additionally, naringenin suppressed tumor growth in two murine OSCC models. The mRNA levels of CD169, interleukin (IL)-12, and C-X-C motif chemokine ligand 10 (CXCL10) in lymph nodes and CTL infiltration in tumors significantly increased following naringenin administration in tumor-bearing mice. These results suggest that CD169^+^ macrophages in lymph nodes are involved in T cell-mediated anti-tumor immunity and could be a prognostic marker for patients with OSCC. Moreover, naringenin is a new potential agent for CD169^+^ macrophage activation in OSCC treatment.

**Supplementary Information:**

The online version contains supplementary material available at 10.1007/s00262-022-03149-w.

## Introduction

Oral squamous cell carcinoma (OSCC) is one of the most common head and neck cancers [1]. The survival rate of patients with OSCC has not improved despite recent progress in diagnostics and treatment. In particular, the prognosis of patients with advanced OSCC remains poor, with a 5-year survival rate of approximately 50% [2]. Thus, new therapeutic approaches must be developed to improve treatment outcomes.

Immune checkpoint inhibitors have recently been approved for the treatment of head and neck cancer and have led to a major turning point for the treatment of OSCC. The immune status of patients is a primary factor for immunotherapy [3]. For instance, the presence and density of tumor-infiltrating lymphocytes (TILs) are important prognostic parameters for head and neck squamous cell carcinoma (HNSCC) [4]. Therefore, to improve patient outcomes by enhancing their anti-tumor immunity, it is necessary to examine novel immune-related anti-tumor factors.

CD169, also called sialoadhesin or sialic acid-binding lectin (Siglec)-1, is a macrophage surface marker belonging to the Siglec family and functions as a sialic acid receptor [5, 6]. CD169 is expressed in macrophages of secondary lymphoid tissues such as lymph nodes (LNs) and spleen [7], especially in macrophages in the subcapsular/medullary sinuses of the LNs and the marginal zone of the spleen. Additionally, CD169 expression is found in certain tissue macrophages in the bone marrow, colon, liver, and lung [7, 8]. Tumor-draining LNs are major lymphatic organs that initiate the anti-tumor immune response [9] and are important for the tumor antigen-specific T cell response [10]. A previous report has shown that CD169^+^ sinus macrophages take up dead tumor cells in the regional lymph nodes (RLNs) and present antigens to activate cytotoxic T lymphocytes (CTLs) [11]. CD43, a glycoprotein expressed on T lymphocytes and myeloid cells, was identified as a counterreceptor for CD169 [12] and suggested to be involved in direct cell–cell interaction between T lymphocytes and CD169^+^ sinus macrophages in human LNs [5]. In addition, the depletion of CD169^+^ macrophages prevents the establishment of anti-tumor immunity [11, 13]. Thus, CD169^+^ sinus macrophages in RLNs are thought to play an important role in establishing anti-tumor immunity [8].

Naringenin, the aglycone form of naringin, is a flavonoid present in grapefruit and other citrus fruits. It is considered to be a safe natural compound [14] and a potential therapeutic agent for a variety of diseases [15–18]. Several reports have also shown that naringenin is a potential anti-tumor agent [19–21]. Naringenin promotes T cell activation and inhibits lung metastasis in a breast cancer resection model [19]. We have previously shown that naringin, the glycosylated form of naringenin, induces CD169 expression and inflammatory cytokine production in LN macrophages [22]. Aglycones are formed by the removal of the glycosyl group from glycosides by the action of intestinal bacteria after the oral administration of a natural product [23, 24]. Therefore, it follows that naringin is also converted into naringenin in the intestine, which is followed by the production of naringenin in vivo, suggesting that the aglycone form (naringenin) is a major active compound in vivo after the oral administration of both naringin and naringenin. Therefore, we speculate that naringenin may also activate CD169^+^ sinus macrophages and, in turn, promote anti-tumor immune responses. However, the effects of naringenin in OSCC remain unclear.

In this study, we analyzed the clinicopathological parameters of resected LN and tumor specimens to investigate the association between CD169^+^ macrophages in RLNs and CTLs in primary tumors. Further, we determined the effects of naringenin on OSCC using tumor-bearing mouse models.

## Materials and methods

### Patient and tissue samples

We evaluated primary tumor and RLN specimens that were resected from 89 patients with OSCC (53 men and 36 women; median age, 71.0 years; range 33–88 years) who underwent radical resection at Kumamoto University Hospital between October 2003 and June 2017. All tumors were categorized according to the TNM classification of the American Joint Committee on Cancer (AJCC; eighth edition) [25], and the degree of differentiation was determined based on the classification guidelines of the World Health Organization [26]. Tissue samples derived from resected specimens were used for analysis. The portion of the primary tumor with the deepest invasion was selected for evaluation. The classification system of the American Head and Neck Society was used to assess the cervical LN levels [27]. LNs were collected mainly from level I–III cases. In metastasis-negative cases, LNs were collected from the site most likely to cause metastasis, considering the location of the primary tumor. In contrast, in cases with LN metastasis, tumor cell-free LNs immediately downstream of the metastatic LNs were collected. The samples were fixed in 10% formalin, embedded, sectioned, and stained with hematoxylin and eosin as previously described [28]. This study was conducted with the approval of the Ethics Committee of Kumamoto University (approval no. RINRI:1427) and in accordance with the guidelines for Good Clinical Practice and the Declaration of Helsinki. The present study was a retrospective analysis, which does not require individual consent; however, all participants had the opportunity to opt out (RINRI1427).

### Immunohistochemical assessment

Protein levels in tissue sections were analyzed via immunohistochemistry, as previously described [29]. Briefly, tissue sections (4-µm thick) were deparaffinized, rehydrated using a graded alcohol series, and incubated with primary antibodies at 4 °C overnight in a humidifying chamber. The primary antibodies used were: mouse anti‐CD169 (clone HSn 7D2; Santa Cruz Biotechnology, Dallas, TX, USA), anti‐CD68 (clone PG‐M1; Nichirei, Tokyo, Japan), and anti‐CD8 (clone C8/144B; Nichirei) for the human sections; and rabbit anti-CD3 (clone SP7, Nichirei), anti-CD4 (clone D7D2A, Cell Signaling Tech., Danvers, MA, USA), and anti-CD8 (clone D4W2Z, Cell Signaling Tech.) for the mouse sections. Then, sequential 60-min incubations with secondary antibodies (Hitofine, Nichirei) and visualization with the DAB substrate system (Nichirei) were performed. All slides were lightly counterstained with hematoxylin for 30 s prior to dehydration and mounting. For double-IHC, HistoGreen substrate (green color; AYS-E109, Eurobio Scientific, Les Ulis, France) was used for peroxidase-based immunostaining.

For cell counting, four non-overlapping high-power fields (× 200 magnification) were randomly selected in tumor areas without necrosis and hemorrhage, and counting was performed using KEYENCE BZ-X800 software (Keyence, Itasca, IL, USA). CD169 scores for expression in LN macrophages were analyzed using a previously described method [30]. Briefly, CD169 staining intensity was scored as 0 (no intensity), 1 (weak intensity that was only detectable in high-power fields), 2 (moderate intensity that was detectable in low-power fields), or 3 (strong intensity). The proportion of CD169^+^ cells was scored as 0 (< 1%), 1 (1–10%), 2 (11–50%), and 3 (> 50%). The intensity and proportion scores were added to provide a CD169 score (range: 0–6), with a low CD169 score defined as 0–4 and a high CD169 score defined as 4.5–6. Two independent observers who had no knowledge of the patients’ clinical status conducted cell counting and scoring in a blinded fashion.

### Cell lines

The NR-S1 and SCC VII cell lines were derived from a spontaneously arising OSCC of C3H mice [31–34]. Cells were cultured in Eagle’s minimum essential medium (Fujifilm Wako Pure Chemical Corporation, Osaka, Japan) supplemented with 10% fetal bovine serum in a humidified atmosphere of 5% CO_2_ at 37 °C. To prepare cells for injection, cultured cells were trypsinized, washed twice, and resuspended in phosphate-buffered saline (PBS).

### Reagents

Naringenin (Tokyo Chemical Industry, Tokyo, Japan) was dissolved in dimethyl sulfoxide (DMSO; Fujifilm Wako Pure Chemical Corporation) at a concentration of 100 mg/mL. A corresponding concentration of DMSO was used for the control group.

### Cell viability assay

To measure cell viability, 5.0 × 10^4^ cells were seeded in 96-well plates in 100 µL of medium, incubated for 24 h, and treated with various concentrations of naringenin (50, 100, 150, and 200 µM). After 24 h, 10 µL of the Cell Counting Kit-8 (Dojindo Laboratories, Kumamoto, Japan) solution, mixed with 100 µL of medium, was added to the 96-well plates and incubated for 1 h at 37 °C. Absorbance was measured at 450 nm using an iMark Microplate Reader (Bio-Rad, Hercules, CA, USA). The absorbance of each well with naringenin was divided by that of the control to determine the relative cell viability (%).

### Mice

Female C3H/HeNCrl mice aged 6 weeks or older were obtained from Charles River (Shiga, Japan). Mice were housed in a temperature-controlled room with a 12-h light/dark cycle under specific-pathogen-free conditions. All animal experiments were approved by the Ethics Committee for Animal Experiments of Kumamoto University (A2020-068) and performed in accordance with the guidelines for animal experiments in the laboratories.

### Murine intraperitoneal naringenin administration

Naringenin was dissolved in 200 µL of PBS and injected intraperitoneally into mice at a concentration of 40 mg/kg. The mice were euthanized 24 h after the injection, and this was followed by the determination of CD169 and cytokine expression in inguinal LNs through real-time polymerase chain reaction analysis.

### RNA in situ hybridization (ISH)

RNAScope Duplex Kit (Advanced Cell Diagnostics, Newark, CA, USA) was used to measure the mRNA expression on paraffin sections.

### Western blotting

LNs isolated from mice were homogenized, and tissue lysates were prepared in NP-40 lysis buffer. A total of 20 mg of each sample was loaded into the wells of a 10% sodium dodecyl sulfate–polyacrylamide gel and transferred onto polyvinylidene fluoride membranes (Millipore, Bedford, MA, USA). The membrane was blocked with 1% skim milk and then incubated with primary antibodies, such as anti-mouse CD169 (ab205104, Abcam), anti-mouse CD68 (ab125212, Abcam), and anti-β-actin (C-2; sc-47778, Santa Cruz Biotechnology). Next, the membrane was incubated with horseradish peroxidase-conjugated secondary anti-IgG antibody (goat anti-rabbit IgG (H + L), 65-6120, Invitrogen) and goat anti-mouse IgG (62-6520, Thermo Fisher Scientific, Waltham, MA, USA). The expression signal was developed using the electrochemiluminescence western blotting detection reagent (Thermo Fisher Scientific).

### Flow cytometry

Cells were treated with FcR-blocking reagent (BioLegend) and reacted with phycoerythrin-labeled anti-CD169 antibody (clone SER-4, Invitrogen), V510-labeled anti-CD11b antibody (clone M1/70, BioLegend), and isotype-matched control antibodies (BioLegend). The stained cell samples were analyzed on a FACSverse flow cytometer (Becton Dickinson, Franklin Lake, NJ, USA) with FACSuite software (Becton Dickinson).

### Electron microscopy

LNs were fixed with 2.5% glutaraldehyde in 0.1 M cacodylate buffer for 1 h and postfixed in 1% osmium tetroxide. After dehydration in a graded series of ethanol solutions with propylene oxide and embedding in Epon 812, ultrathin sections were cut with an ultratome, stained with uranyl acetate and lead citrate, and observed using a Hitachi H-7700 electron microscope (Hitachi, Tokyo, Japan).

### Real-time quantitative polymerase chain reaction (RT-qPCR)

Total RNA was isolated using a FastGene RNA Basic Kit (Nippon Genetics, Tokyo, Japan). RNA was reverse-transcribed using the ReverTra Ace qPCR RT Kit (Toyobo, Osaka, Japan). RT-qPCR was performed using Thunderbird SYBR qPCR Mix (Toyobo) on CFX Connect (Bio-Rad, Hercules, CA, USA). For quantification, the mRNA levels were normalized to those of β-actin. All primers used for CD169, interleukin (IL)-12, and C-X-C motif chemokine ligand 10 (CXCL10) analyses are listed in Table S1.

### Murine allograft model

Naringenin was administered to mice, and NR-S1 cells (2.0 × 10^7^) and SCC VII cells (1.0 × 10^7^) were subcutaneously inoculated after 4 d into both sides of the shaved back of the mice. Naringenin was intraperitoneally administered at a concentration of 40 mg/kg every 4 days. In the control group, PBS containing DMSO at a concentration corresponding to that in the naringenin group was intraperitoneally administered. Tumor volumes were monitored and measured using calipers. Tumor volumes were estimated using the formula: length × width^2^ × *π*/6 [35]. The mice were euthanized, the tumors and inguinal LNs were removed, and inguinal LN volumes were estimated using the formula *π*/6 × (length × width)^3/2^ [36].

### Statistical analyses

The cumulative survival rates were compared between groups using the log-rank test. Differences in mean values between groups were analyzed using the Mann–Whitney *U* test, whereas differences in mean values among multiple groups were analyzed using one-way ANOVA followed by the Bonferroni/Dunn test. The correlation between the CD169 score and number of CD8-positive cells was assessed using Spearman’s rank correlation. All *p* values were based on two-tailed statistical analyses; *p* values < 0.05 were considered statistically significant. Multivariate analysis was performed using JMP 9 software (SAS Institute Inc., Cary, NC, USA), and the other analyses were performed using Statcel 4 software (OMS Publishing Inc., Saitama, Japan).

## Results

### Correlation between CD169 expression in LN macrophages and CD8^+^ T cell infiltration in the primary tumors of patients with OSCC

**Fig. 1:**
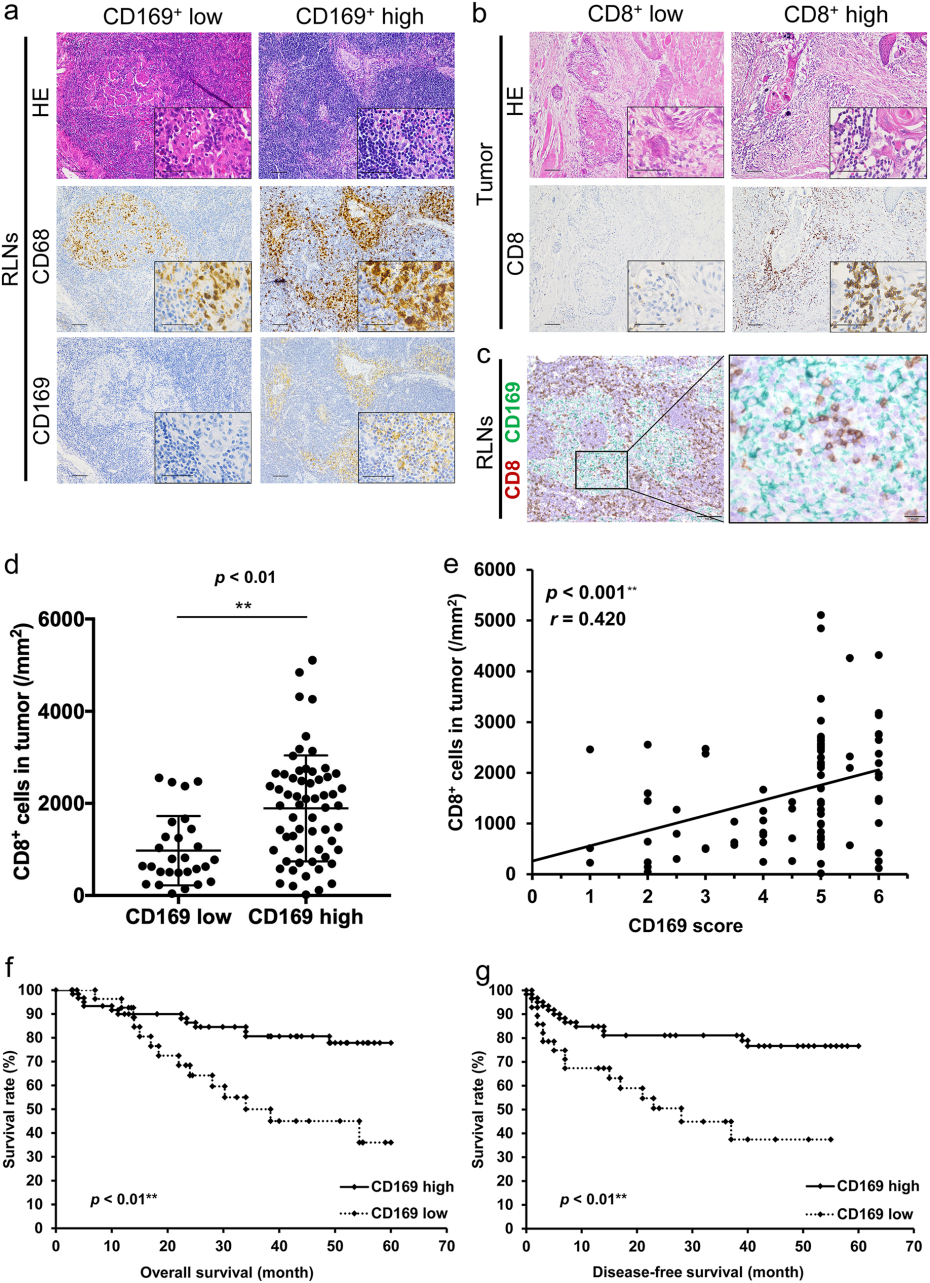
Increased CD169^+^ expression in macrophages in the regional lymph nodes (RLNs) of oral squamous cell carcinoma (OSCC) patients correlated with enhanced CD8^+^ T cell infiltration in the primary tumor and with better prognosis. Hematoxylin and eosin staining and immunostaining of **a** CD68^+^ and CD169^+^ macrophages in the sinus areas of the RLNs and **b** CD8^+^ T cells in primary oral tumor tissues of patients. Representative images of the tissue sections with high or low numbers of CD169^+^ macrophages (**a**) and high or low numbers of CD8^+^ cells (**b**) are shown. Larger images were taken under lower magnification; scale bar: 100 µm. The lower right insets show images from higher magnifications; scale bar: 50 µm. **c** Double IHC using anti-CD8 antibody (brown) and CD169 antibody (green) was performed. Scale bar; 100 µm (left), 20 µm (right). **d**, **e** Analyses of the correlation between the number of CD8^+^ T cells in the tumor tissues and the CD169 score in the RLNs. For **d**, **e** *p* values were calculated using the Mann–Whitney *U* test (**d**) and Spearman’s rank correlation (**e**). Values represent the mean ± SD. **p < 0.01. **f**, **g,** Kaplan–Meier curves for overall survival (OS) (**f**) and disease-free survival (DFS) (**g**) of 89 OSCC patients based on CD169 expression in the RLN macrophages

We used IHC to evaluate the expression of CD169 and CD68 in RLNs and CD8 in tumor tissues obtained from patients with OSCC who underwent radical resection. As in previous reports [30, 37], the total numbers of CD68^+^ macrophages in the LNs were similar among all patients (Fig. 1a). However, the proportion of CD169^+^ cells and intensity of CD169 staining differed greatly among patients (Fig. 1a). Then, we counted the number of CD8^+^ T cells in the primary tumor and analyzed their correlation with the patients’ clinicopathological factors and CD169 expression in LNs. The number of CD8^+^ cells differed greatly among patients (Fig. 1b). Double-IHC of CD8 and CD169 in LNs showed the direct cell–cell interaction between CD169^+^ macrophages and lymphocytes in the sinus area (Fig. 1c). Regression analysis revealed a positive correlation between the CD169 score from the patients’ LNs and density of CD8^+^ T cells in the primary tumors (Fig. 1d, e). Similar results were obtained from the correlation analysis (Table S2).

### Correlation between levels of CD169 expression in RLN sinus macrophages and patient survival

To assess the correlation between CD169 expression levels in RLN sinus macrophages and patient survival, we analyzed overall survival (OS) and disease-free survival (DFS) of the 89 patients with OSCC using the Kaplan–Meier method. High expression levels of CD169 in RLN macrophages significantly correlated with better OS (log-rank test, *p* = 0.002, Fig. 1f) and DFS (log-rank test, *p* = 0.001, Fig. 1g). Multivariate analysis showed a significant correlation between OS and high expression levels of CD169 in RLN macrophages (Table 1). Collectively, our data indicate that the expression level of CD169 is a potential prognostic marker for patients with OSCC.

**Table 1:** Univariate and multivariate Cox regression analyses of potential prognostic factors for overall survival in patients with OSCC (*n* = 89)

Characteristics	*n*	Univariate analysis	Multivariate analysis	*p* value
		Log-lank *p* value	Hazard ratio (95% CI)	
Age (years)				
≤ 65	34	0.491	ND	ND
> 65	55			
Gender				
Male	53	0.112	ND	ND
Female	36			
Primary site				
Tongue	37	0.545	ND	ND
Mandible	24			
Oral floor	11			
Buccal mucosa	10			
Maxilla	7			
Clinical T category				
T1, T2	44	0.458	ND	ND
T3, T4	45			
Clinical N category				
N0	27	0.959	ND	ND
N ≥ 1	62			
Pathological T category				
T1, T2	46	0.300	ND	ND
T3, T4	43			
Pathological N category				
N0	50	0.071	1.795 (0.818–4.068)	0.144
N ≥ 1	39			
Differentiation				
Well	51	0.468	ND	ND
Poor, moderate	38			
CD8^+^ cells/mm^2^ in the tumor				
> 1588	41	0.864	ND	ND
≤ 1588	48			
CD169 score				
High	61	0.002*	3.009 (1.374–6.692)	0.006*
Low	28			

OSCC, oral squamous cell carcinoma; CI, confidence interval; ND, not done; LN, lymph node. The average number of CD8^+^cells/mm^2^in the tumor was 1588

*Statistically significant results

### Effects of naringenin on the proliferation of OSCC cell lines

To determine the effects of naringenin on the proliferation of OSCC, we performed an in vitro experiment using two cell lines, NR-S1 and SCC VII. Different concentrations of naringenin had no significant effects on the viability of either NR-S1 or SCC VII cells (Fig. S1a and b). These results indicate that naringenin has little cytotoxic effect on OSCC cells.

### Effects of naringenin on LN macrophage activation in mice

**Fig. 2:**
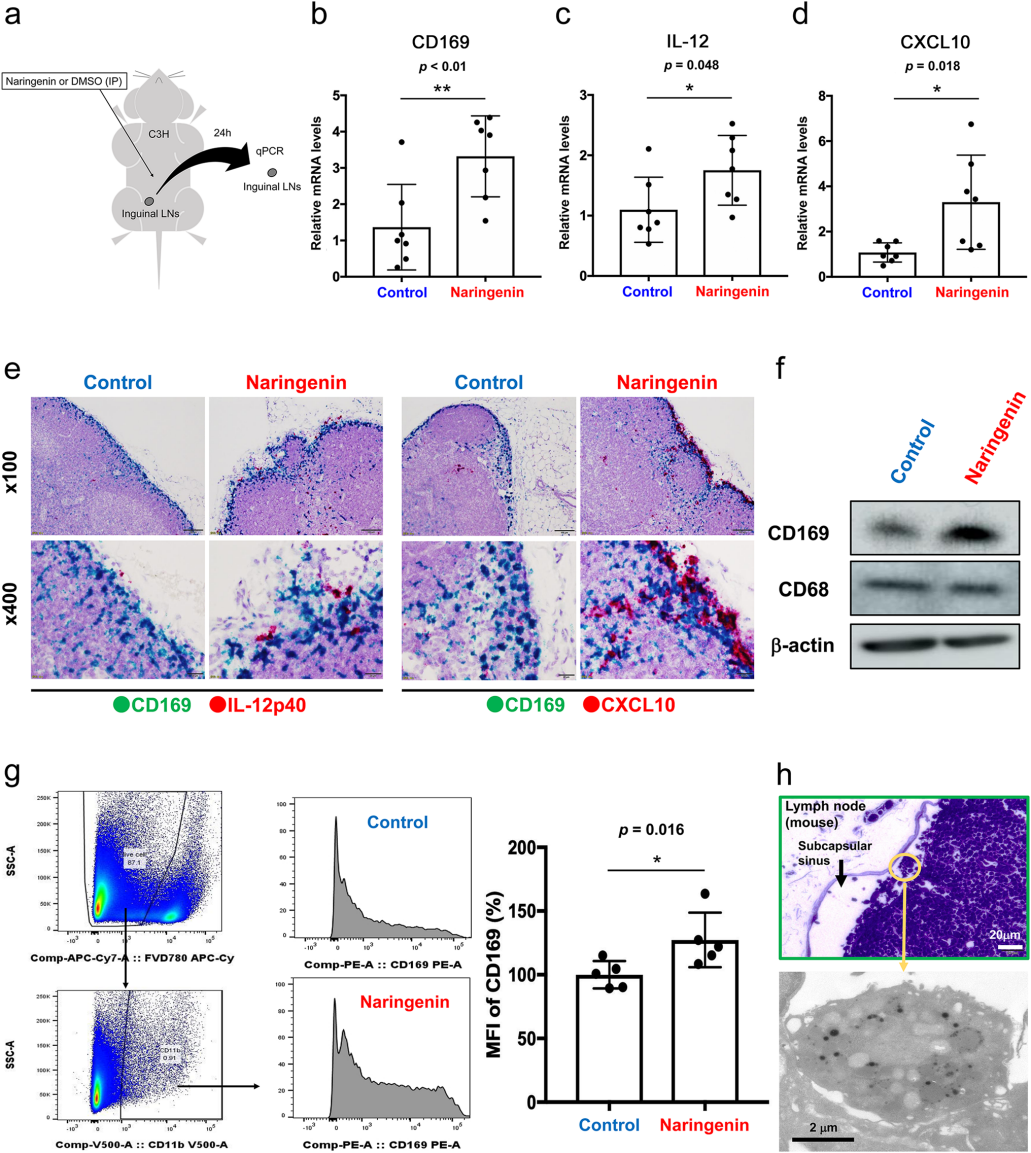
Naringenin upregulates CD169 and other factors that induce cytotoxic T cell activation. **a** Schematic illustration of the in vivo study using a mouse allograft model. **b**–**d** Expression levels of CD169, IL-12, and CXCL10 in the right inguinal lymph nodes of mice following intraperitoneal injection with 200 µL of naringenin (40 mg/kg) or DMSO in mice (*n* = 7 per group) were determined by RT-qPCR. Relative mRNA levels to non-treatment are shown. **e** ISH of CD169 (green), IL-12p40 (red), and CXCL10 (red) in lymph nodes is presented. IL-12p40 and CXCL10 expression was increased by naringenin treatment. IL-12p40 and CXCL10 were also detected in CD169-negative cells of the medullary area and these positive cells seemed to be dendritic cells. Scale bar; 100 µm (× 100), 20 µm (× 400). **f** Western blot analysis of CD169, CD68 (marker for macrophage), and β-actin using lymph node lysates is presented. **g** Flow cytometry of lymph node cells and mean fluorescent intensity (MFI) of CD169 expression in CD11b^+^ cells are presented (*n* = 5). Fixable Viability Dye Fluor (FVD) 780 was used for depleting the dead cells. **h** Upper panel shows toluidine blue staining of semi-thin section of murine lymph node dissected from mice into which tumor cells were pre-injected subcutaneously. Lower panel shows electron microscopy of macrophages that phagocytosed apoptotic bodies in the phagosomes. The values were normalized to β-actin. Values represent the mean ± SD. *p < 0.05 and **p < 0.01

We next examined the effect of naringenin on LN macrophage activation in mice using the methods shown in Fig. 2a. The intraperitoneal administration of naringenin increased the transcription of genes encoding CD169, IL-12p40, and CXCL10 in inguinal LNs (Fig. 2b–d). ISH revealed that naringenin induced the overexpression of IL-12p40 and CXCL10 mRNA in CD169-expressing sinus macrophages (Fig. 2e). Western blot analysis of whole lymph node lysate showed that naringenin elevated CD169 protein levels in LNs (Fig. 2f). Increased surface CD169 expression on macrophages was confirmed by flow cytometry (Fig. 2g). The engulfment of apoptotic cells by sinus macrophages was confirmed by electron microscopy (Fig. 2h). As IL-12 and CXCL10 augment the function of CD8^+^ T cells, these results suggest that naringenin induces cytotoxic T cell activation via CD169^+^ macrophages in vivo.

### Suppressive effects of naringenin on tumor growth in vivo

**Fig. 3:**
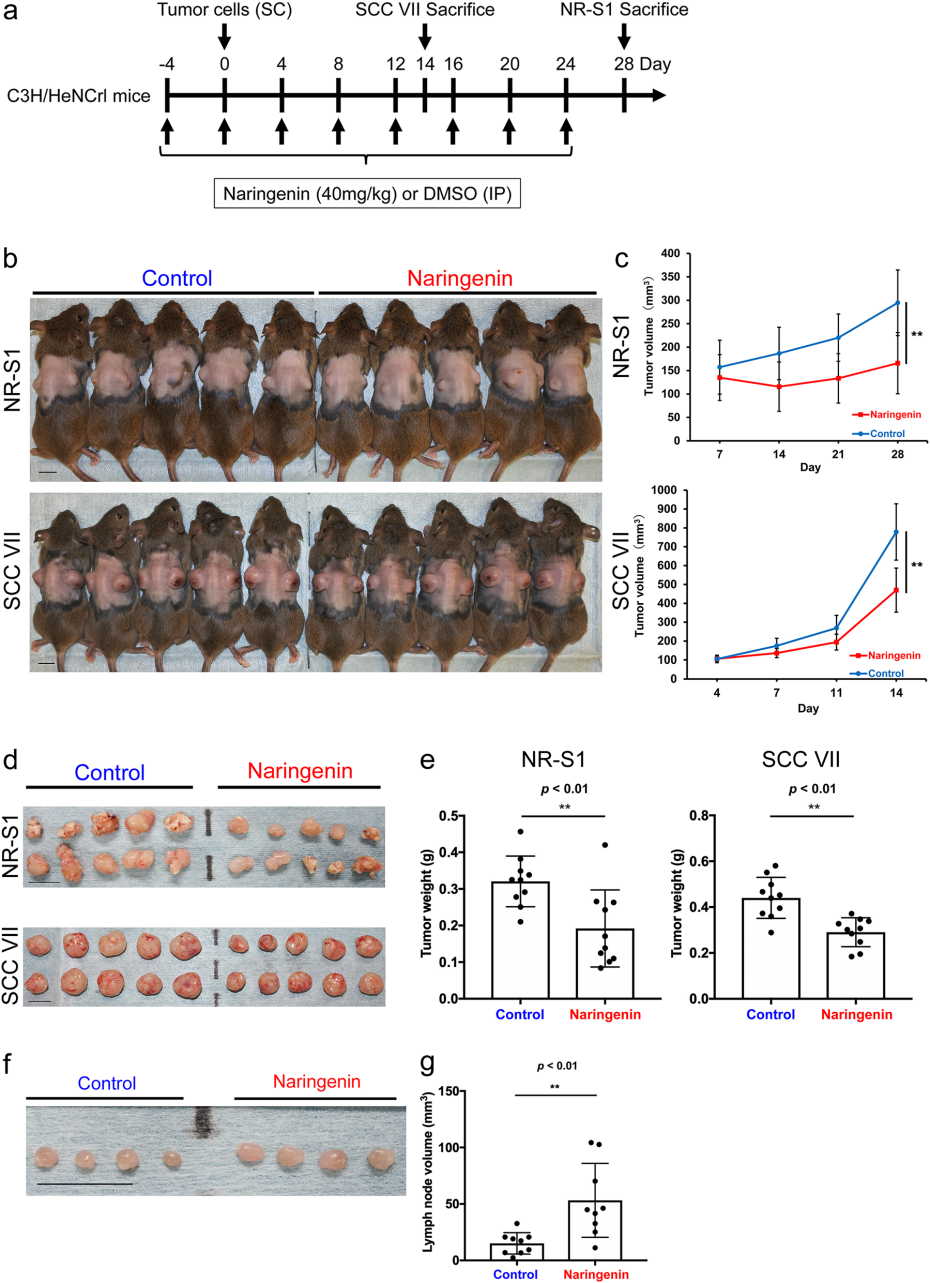
Naringenin suppresses primary tumor growth in vivo. **a** Experimental protocol for assessing anti-tumor activity of naringenin in vivo. The schedule of tumor cell injections and naringenin injections is shown. SC, subcutaneous; IP, intraperitoneal. **b** Images of mouse tumors indicating suppressive effects of naringenin on tumor growth. **c** Effect of naringenin on volumes of tumors grafted using two OSCC cell lines. **d**,** e** Tumor sizes and weights at the end of the experiment. Scale bar, 10 mm. Values represent the mean ± SD (*n* = 10 tumors per group). **p < 0.01. **f**, **g** Representative images of inguinal lymph nodes removed from allograft models. Inguinal lymph node volumes were measured at the end of the experiment. Nine mice (one lymph node per mouse) were used in each group. Scale bar, 10 mm. Values represent the mean ± SD. **p < 0.01

We evaluated the anti-tumor effects of naringenin in an allograft model. As shown in the protocol in Fig. 3a, the intraperitoneal administration of naringenin was performed on mice with the grafted tumors. We confirmed that naringenin significantly suppressed the tumor growth of both cell lines (*p* < 0.01, Fig. 3b, c and Fig. S2a and b). Direct weight measurement of the tumors from five mice per group (Fig. 3b, d) verified the anti-tumor effects of naringenin (*p* < 0.01, Fig. 3e). The inguinal LNs resected from the mice were significantly swollen in the naringenin group (*p* < 0.01, Fig. 3f, g). These results suggest that naringenin suppresses tumor growth by activating CD169^+^ sinus macrophages in RLNs.

### Immunohistochemical analysis of mouse tumors

**Fig. 4:**
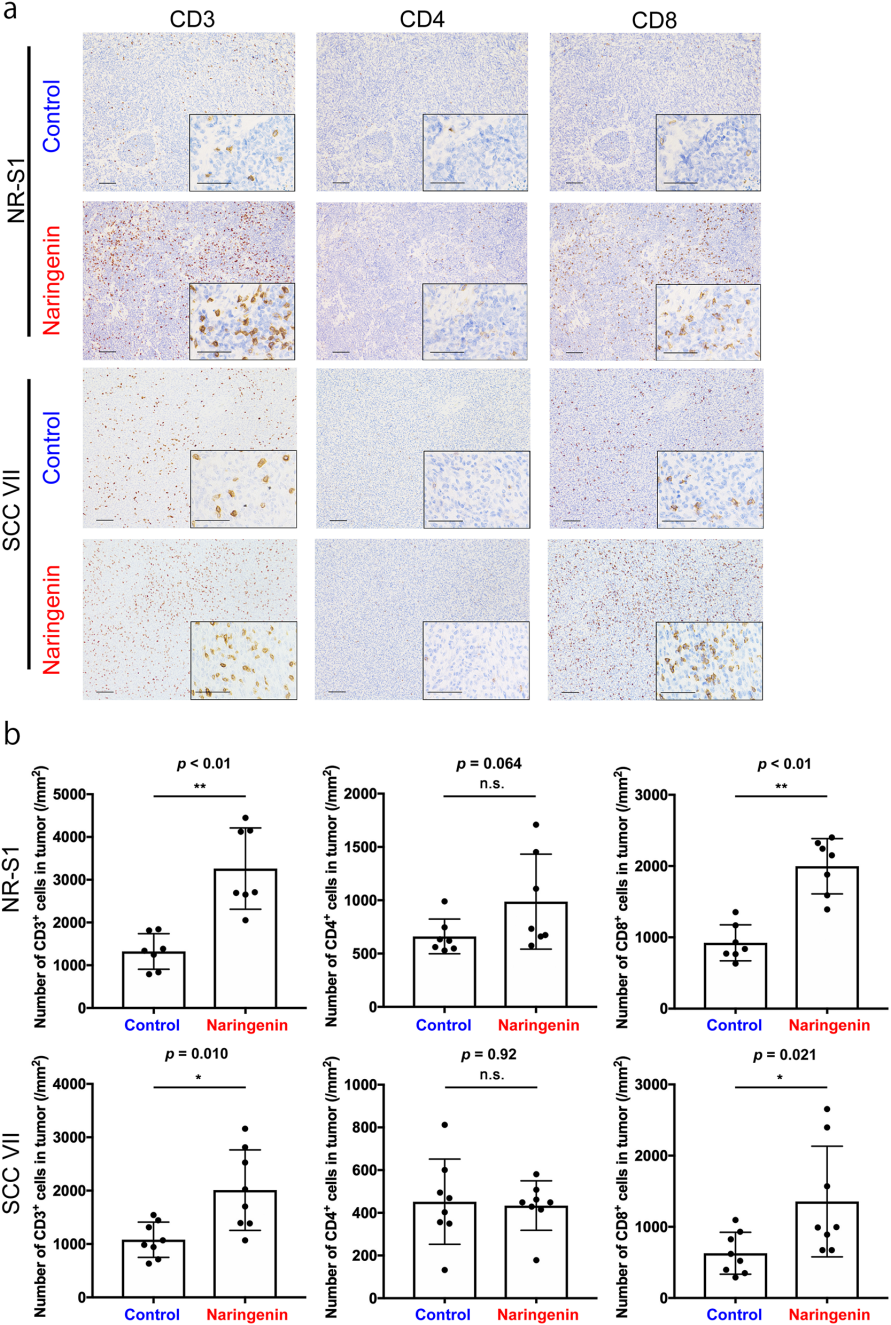
Naringenin enhances CD8^+^ T cell infiltration in the primary tumor tissue. **a** Representative images of immunohistochemical analyses for CD3, CD4, and CD8 on NR-S1 and SCC VII allograft models. Larger images were taken at lower magnification; scale bar: 100 µm. The lower right insets show images taken at higher magnification; scale bar: 50 µm. **b** The number of CD3^+^, CD4^+^, and CD8^+^ cells per mm^2^ (*n* = 7 for NR-S1, *n* = 8 for SCC VII per group). Values represent the mean ± SD. n.s., no significant difference; *p < 0.05 and **p < 0.01

Significantly higher CD3^+^ T cell counts were found in the tumor tissues of mice in the naringenin group than in those of mice in the control group (Fig. 4a). In particular, the CD8^+^ T cell counts significantly increased following naringenin administration, reflecting the activation of anti-tumor immunity (Fig. 4b). These results suggest that naringenin also activates CD8^+^ cells via the activation of CD169^+^ sinus macrophages in RLNs, leading to the suppression of tumor growth. These findings open the possibility of a new treatment option that stimulates anti-tumor immunity in OSCC.

## Discussion

In this study, we demonstrated that high expression levels of CD169^+^ in RLN macrophages positively correlated with the number of CD8^+^ T cells infiltrating the primary tumor and strongly associated with improved OS and DFS rates. Our univariate and multivariate analyses also revealed that a high CD169^+^ expression level was an independent predictor of improved prognosis in patients with OSCC. Furthermore, we showed that naringenin suppressed tumor growth by upregulating CD169 expression in the host LNs and activating anti-tumor immunity in a tumor-bearing mouse model. Generally, tumor size and nodal status (TN) are the most significant prognostic factors in oral cancer [38, 39], but no significant difference was found in TN staging in this study. Although the correlation between locoregional recurrence or distant metastasis and CD169^+^ expression level was not investigated in this study, it is suggested that the expression levels of CD169^+^ in RLN macrophages after the end of treatment prevent locoregional recurrence and distant metastasis.

Previous studies have shown that CD169^+^ macrophages in RLNs and CD8^+^ lymphocytes in tumors are strongly correlated in various cancer types [5, 6, 30, 40, 41]. According to a previous animal study, the subcutaneous injection of dead tumor cells into mice expands the population of tumor antigen-specific CD8^+^ T cells in the draining LNs, protecting these mice from future progression of live tumors [11]. Furthermore, the selective depletion of CD169^+^ macrophages in mice cancels the protective effects of a dead tumor cell vaccine [8]. These reports suggest the ability of CD169^+^ macrophages to activate tumor antigen-specific CD8^+^ T cells. A growing number of studies on TILs show that CD8^+^ T cells are key components of anti-tumor immunity [42]. Given the correlation between TILs and patient survival for various types of cancers including OSCC [43–45], our findings suggest that CD169^+^ macrophages are closely related to CD8^+^ T cell-mediated activation of anti-tumor immunity.

Our in vitro experiments revealed that naringenin has little direct cytotoxicity in mouse oral cancer cell lines. The results of RT-qPCR analyses showed that naringenin treatment resulted in the upregulation CD169 and cytokine expression in the LNs of mice. A previous report has shown that certain natural compounds, such as naringin, promote CD169 expression in LNs and induce an anti-tumor phenotype (M1-like phenotype) in macrophages, which are similar to the effects of interferon (INF)-α in vivo [22]. Type I IFNs, such as IFN-α, induce an anti-tumor phenotype in macrophages [46]. When naringin is orally administered to humans, carbohydrate chains are degraded by the intestinal bacteria and naringin is converted to naringenin; thus, we used naringenin in this study [42]. Naringenin, like naringin, may inhibit tumor progression by inducing CD169^+^ and M1-like macrophages and triggering CTL activation. In fact, our in vivo experiments showed that naringenin administration upregulated CD169 expression in the LNs, suppressed tumor growth, and promoted CTL infiltration in tumors. Swelling of the inguinal LNs of the mice in the naringenin group was presumed to be a result of naringenin-induced activation of immunity. Our findings suggest that augmenting anti-tumor immune responses through the enhancement of antigen presentation in RLNs is an effective therapeutic strategy for OSCC.

One major limitation of this study is that although a previous study has shown that CD169^+^ macrophages phagocytose dead tumor cells and cross-present tumor antigens to CD8^+^ T cells for activation in an animal model [11], we were unable to confirm the direct link between dead OSCC cells and CD8^+^ T cell activation in our model. Furthermore, our study focused solely on LN CD169^+^ macrophages; we need to verify the effects of other antigen-presenting cells, such as dendritic cells, in primary tumors and LNs on CTL activation.

In recent years, immunotherapy for cancer has received a great deal of attention. Medicines that target the programmed cell death 1 (PD-1)–programmed death-ligand 1 pathway, also known as immune checkpoint inhibitors, have dramatically changed the treatment of various types of cancers, including melanoma [47, 48]. The benefits of PD-1 inhibitors, such as nivolumab and pembrolizumab, to the treatment of recurrent or metastatic HNSCC have been demonstrated [49, 50], and they have significantly changed the treatment strategy for OSCC. Moreover, it is becoming increasingly clear that host anti-tumor immunity is closely linked to the therapeutic effects of these immunotherapies [3, 51]. In line with this, our results indicate the involvement of CD169^+^ macrophages in the regulation of anti-tumor immunity against OSCC. Thus, our study provides new insights into anti-tumor immunity in HNSCC, including OSCC.

To the best of our knowledge, this is the first study to demonstrate the clinical significance of CD169^+^ macrophages in the RLNs of patients with OSCC. Furthermore, we are the first to show naringenin-induced activation of anti-tumor immunity via the enhancement of CD169 expression in macrophages in LNs and the promotion of CTL infiltration into OSCC tumors. Moreover, our results suggest that CD169^+^ expression in LN macrophages is a prognostic marker for OSCC and that naringenin is a novel potential agent for OSCC treatment.

### Supplementary Information

Below is the link to the electronic supplementary material.

Supplementary file1 (PDF 338 KB)

## Data Availability

All data generated or analyzed during this study are included in this published article.

## Abbreviations

AJCC: American Joint Committee on Cancer
CTL: Cytotoxic T lymphocytes
HNSCC: Head and neck squamous cell carcinoma
LN: Lymph node
OSCC: Oral squamous cell carcinoma
RLN: Regional lymph node
TILs: Tumor-infiltrating lymphocytes
