# Supplementary material for: Naringenin potentiates anti-tumor immunity against oral cancer by inducing lymph node CD169-positive macrophage activation and cytotoxic T cell infiltration
Source: Cancer Immunol Immunother. 2022 Jan 19;71(9):2127–39. doi: 10.1007/s00262-022-03149-w (PMC9374624; doi:10.1007/s00262-022-03149-w)
Supplement: Supplementary file 1 — Supplementary file1 (PDF 338 KB) [file 262_2022_3149_MOESM1_ESM.pdf]

Supplementary information

Cancer Immunology, Immunotherapy (Submitted in 2021) Kawaguchi et. al.

Table S1

Primer name	Primer sequence	
	Sense	Antisense
CD169	5′-ACCTGCCCAGCTAACTCTCA-3′	5′-CCTTCCAGCAGAAGTTCCAG-3′
IL-12	5′-AGCACCAGCTTCTTCATCAG-3′	5′-CTTGAGGGAGAAGTAGGAATGG-3′
CXCL10	5′-CCAAAGGGATGAGAAGTTCC-3′	5′-TCCACTTGGTGGTTTGCTAC-3′

Supplementary Table S1 Primers used for RT-qPCR

Table S2

Characteristics	Total	CD169 expression		*p*-value
		Low n (%)	High n (%)	
Age (years)				
Median	71.0	71.0	69.5	
Range	33-88	33-88	35-86	
≤65	34	11 (39.3)	23 (37.7)	0.887
>65	55	17 (60.7)	38 (62.3)	
Gender				
Male	53	17 (60.7)	36 (59.0)	0.880
Female	36	11 (39.3)	25 (41.0)	
Primary site				
Tongue	37	10 (35.7)	27 (44.3)	0.180
Mandible	24	6 (21.4)	18 (29.5)	
Oral floor	11	6 (21.4)	5 (8.2)	
Buccal mucosa	10	2 (7.1)	8 (13.1)	
Maxilla	7	4 (14.3)	3 (4.9)	
Clinical T category				
T2	44	12 (42.9)	32 (52.5)	0.400
T3, T4	45	16 (57.1)	29 (47.5)	
Clinical N stage				
N0	27	8 (28.6)	19 (31.1)	0.806
N ≥ 1	62	20 (71.4)	42 (68.9)	
Pathological T category				
T2	46	11 (39.3)	35 (57.4)	0.113
T3, T4	43	17 (60.7)	26 (42.6)	
Pathological N category				
N0	50	14 (50.0)	36 (59.0)	0.426
N ≥ 1	39	14 (50.0)	25 (41.0)	
Differentiation				
Well	51	18 (64.3)	33 (54.1)	0.367
Poor, Moderate	38	10 (35.7)	28 (45.9)	
$CD8^+$ cells/mm^2 in the tumor				
>1588	41	6 (21.4)	35 (57.4)	0.002*
≤1588	48	22 (78.6)	26 (42.6)	

*Statistically significant results. Abbreviation: OSCC, oral squamous cell carcinoma; LN, lymph node. The average number of $CD8^+$ cells/mm^2 in the tumor was 1588.

Supplementary Table S2 Correlation between CD169 expression and clinicopathological factors in 89 patients with OSCC

Supplementary Fig. 1

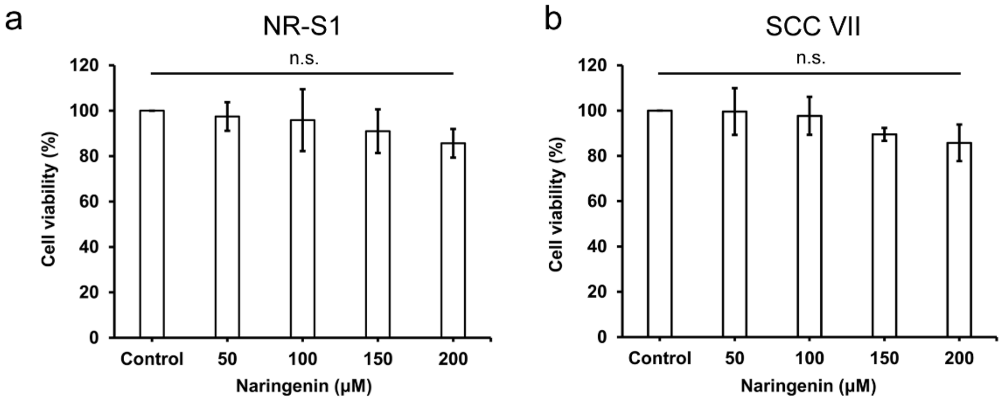

Supplementary Fig. S1 The effects of naringenin on the proliferation of OSCC cell lines *in vitro*

a, b, Viability of NR-S1 and SCC VII cells treated with naringenin. Cells were incubated with various concentrations of naringenin for 24 h. Each experiment was repeated three times. n.s., not significant; data were analyzed through one-way analysis of variance (ANOVA) followed by the Bonferroni/Dunn test.

Supplementary Fig. 2

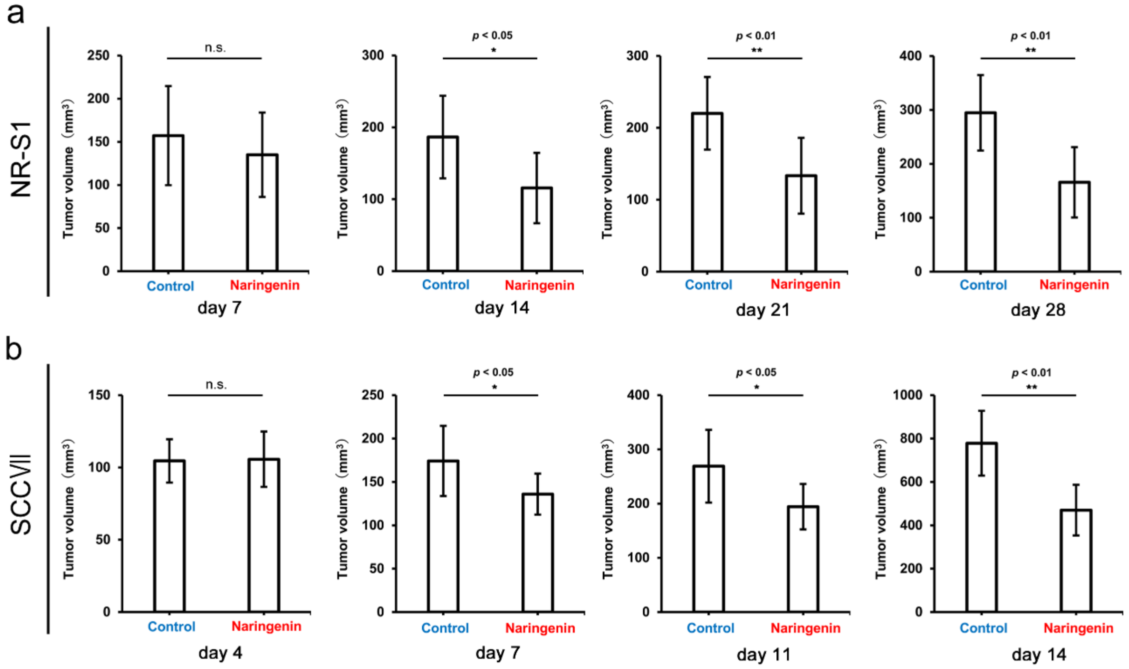

Supplementary Fig. S2 The effect of naringenin on tumor growth each day *in vivo*

a, b, Naringenin significantly suppressed tumor growth of both cell lines. Data were analysed through the Mann-Whitney U test. Values represent the mean ± SD. *$p < 0.05$, **$p < 0.01$.
